# Amygdala neurocircuitry at the interface between emotional regulation and narcolepsy with cataplexy

**DOI:** 10.3389/fnins.2023.1152594

**Published:** 2023-04-21

**Authors:** Haniyyah Sardar, Andrea N. Goldstein-Piekarski, William J. Giardino

**Affiliations:** ^1^Department of Psychiatry and Behavioral Sciences, Stanford University School of Medicine, Stanford, CA, United States; ^2^Center for Sleep and Circadian Sciences, Stanford University School of Medicine, Stanford, CA, United States; ^3^Wu Tsai Neurosciences Institute, Stanford University School of Medicine, Stanford, CA, United States

**Keywords:** amygdala, extended amygdala, narcolepsy, cataplexy, sleep, orexin, hypocretin, bed nucleus of stria terminalis

## Abstract

Narcolepsy is a sleep disorder characterized by chronic and excessive daytime sleepiness, and sudden intrusion of sleep during wakefulness that can fall into two categories: type 1 and type 2. Type 1 narcolepsy in humans is widely believed to be caused as a result of loss of neurons in the brain that contain the key arousal neuropeptide Orexin (Orx; also known as Hypocretin). Patients with type 1 narcolepsy often also present with cataplexy, the sudden paralysis of voluntary muscles which is triggered by strong emotions (e.g., laughter in humans, social play in dogs, and chocolate in rodents). The amygdala is a crucial emotion-processing center of the brain; however, little is known about the role of the amygdala in sleep/wake and narcolepsy with cataplexy. A collection of reports across human functional neuroimaging analyses and rodent behavioral paradigms points toward the amygdala as a critical node linking emotional regulation to cataplexy. Here, we review the existing evidence suggesting a functional role for the amygdala network in narcolepsy, and build upon a framework that describes relevant contributions from the central nucleus of the amygdala (CeA), basolateral amygdala (BLA), and the extended amygdala, including the bed nucleus of stria terminalis (BNST). We propose that detailed examinations of amygdala neurocircuitry controlling transitions between emotional arousal states may substantially advance progress in understanding the etiology of narcolepsy with cataplexy, leading to enhanced treatment opportunities.

## Introduction

### The amygdala and extended amygdala

The amygdala and accessory regions referred to as the *extended amygdala*, are key emotional centers in the brain, which have classically been studied in the context of motivated actions, defensive behaviors, and fear learning. Anatomical, molecular, physiological, and behavioral studies have delineated functionally distinct subregions of the amygdala, including the basolateral, central, and medial subregions (BLA, CeA, and MeA, respectively). In addition to the classical amygdala structures, the bed nucleus of the stria terminalis (BNST) is a heterogeneous forebrain region with multiple neuronal subpopulations that has also been characterized as part of the *extended amygdala* (Giardino and Pomrenze, 2021; Ortiz-Juza et al., 2021). Like the CeA, the BNST is composed primarily of neurons that utilize the fast inhibitory neurotransmitter GABA. The vast majority of these GABAergic neurons also synthesize and release varying combinations of several neuropeptide modulators, although such multiplex forms of neural transmission remain largely uncharacterized.

Here, our abbreviation of AMY refers to structures of the classical amygdala (BLA, CeA, and MeA), as well as the *extended amygdala* (BNST), as both are critical for behavioral responding to emotionally salient stimuli and consolidation of emotional memories (Phelps and LeDoux, 2005; Lebow and Chen, 2016). While the AMY has historically been primarily studied in the context of threat avoidance and fear conditioning (LeDoux, 2000; Phelps and LeDoux, 2005), extensive research has linked the AMY not only with negative emotional states of anxiety and aversion, but with positive emotional states of approach and reward as well (Janak and Tye, 2015; Ch'ng et al., 2018).

More recently, experimental manipulations of CeA and BNST neurons also revealed their abilities to potently modulate transitions between sleep and wakefulness (Kodani et al., 2017; Ma et al., 2019). These sparse reports extend the functions of AMY neurons far beyond their canonical roles as detectors and mediators of affective behavioral states (Matei et al., 2022). In fact, they likely represent only “the tip of the iceberg” in terms of the multifaceted contributions of AMY circuits to arousal regulation, and in particular, the relationships between emotional regulation and arousal (wakefulness) in sleep disorders such as narcolepsy.

### Narcolepsy and cataplexy

Narcolepsy is a sleep disorder impacting ~4 million people across the globe that is characterized by excessive daytime sleepiness and abnormal episodes of sleep that intrude upon normal wakefulness (Bassetti et al., 2019). Human patients with narcolepsy can experience sleep-like episodes at any period throughout the day, often coupled with hypnopompic or hypnagogic hallucinations (hallucinations that occur while waking up or while falling asleep, respectively; Scammell, 2015; Mahoney et al., 2019). Furthermore, approximately half of people with narcolepsy are estimated to experience symptoms of rapid eye movement (REM) sleep behavior disorder, in which normal muscle atonia during REM fails and they may physically act out the behaviors ongoing in their dreams (Mahoney et al., 2019). One popular interpretation of narcolepsy is that it is an inappropriate intrusion of REM sleep into wakefulness. Other interpretations exist—for a summary, see Adamantidis et al. (2020). Previous studies in humans and animals have identified genetic markers linked to increased risk of narcolepsy (Scammell, 2015), and the neuroimmune hypothesis of narcolepsy has been described in detail elsewhere (Mahoney et al., 2019). Those topics are beyond the scope of this article, and we refer the reader to other expert reviews.

Categorized into two types, narcolepsy type 1 (NT1) presents with more severe symptoms and is accompanied by *cataplexy* (the sudden loss of muscle tone, often triggered by strong emotional stimuli; AASM, 2014). In contrast, narcolepsy type 2 (NT2) has less severe symptoms and is not coupled with cataplexy. For these reasons, our review will focus exclusively on NT1 rather than NT2, although there is scant literature suggesting that lesions of the amygdala can generate symptoms of NT2 (Kim et al., 2018).

NT1 in humans is strongly associated with the loss of a specific subpopulation of neurons in the lateral hypothalamus (LH) containing the wake-promoting neuropeptide Orexin (Orx; also known as Hypocretin; Thannickal et al., 2000). Correspondingly, NT1 patients display lower levels of Orx in the cerebrospinal fluid (Nishino et al., 2000) as well as of Orx mRNA and peptide in the brain (Peyron et al., 2000). The Orx peptide system is critical for stabilizing wakefulness (arousal) and Orx-LH neurons project broadly throughout the brain to exert excitatory physiological effects *via* signaling of the G-protein-coupled Orexin receptors type 1 and 2 (OX_1_, OX_2_; Li et al., 2017). Orx activity is essential not only for initiating and maintaining wakefulness, but also for many motivated behaviors such as avoiding threatening stimuli or approaching rewarding stimuli (Giardino and de Lecea, 2014; Mahler et al., 2014; Giardino et al., 2018). NT1 has been identified in other animals as well, with canine NT1 being caused by a mutation in the OX_2_ receptor gene (Lin et al., 1999). NT1 can also be modeled using genetic manipulations of the Orx system, as discussed later.

During episodes of cataplexy, awake and alert subjects can lose muscle tone and eventually experience muscle paralysis and fall into a REM sleep-like state, and these transitions can be detectable by loss of muscle activity on electromyogram recordings (EMG). Frequently, cataplexy is triggered by individuals experiencing powerful positive emotional stimuli, such as laughter or social interactions (Schiappa et al., 2018). In rare cases, it may be triggered by sexual intercourse (Poryazova et al., 2009a). Occasionally, negative emotional stimuli such as anger or frustration can also initiate cataplexy (Schiappa et al., 2018). It has been shown in mouse models of narcolepsy that both appetitive/rewarding and aversive stimuli can increase cataplexy (Espana et al., 2007; Clark et al., 2009; Morawska et al., 2011).

**Figure 1:**
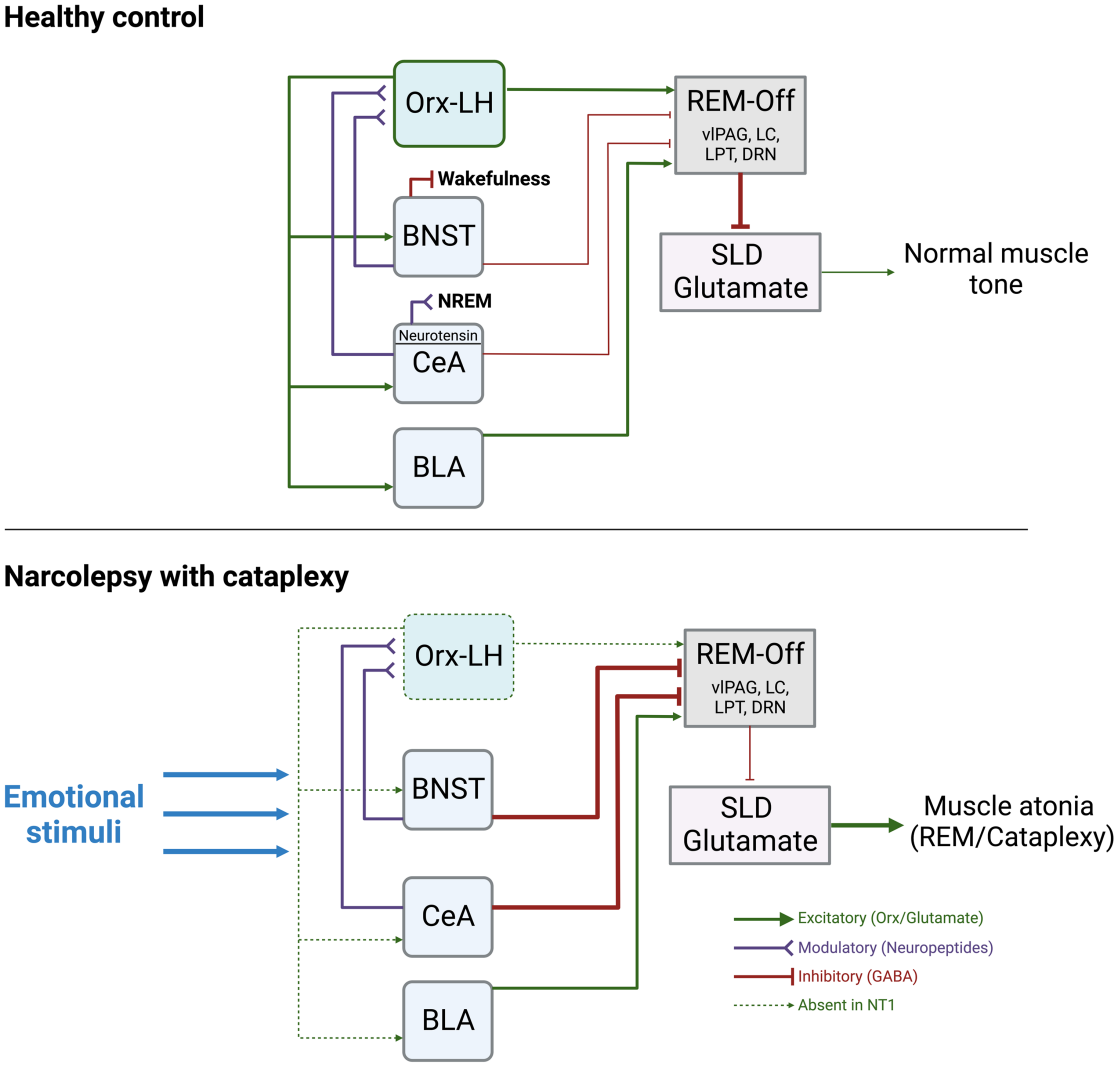
Summary of the known functional connections between AMY, Orx-LH neurons, REM-off promoting neurons, and the SLD. The thickness of the lines indicates hypothesized strengths and activity levels of connections. Under normal conditions, stimulation of BNST-GABA neurons can trigger wakefulness, and stimulation of CeA-Neurotensin neurons can increase NREM sleep. The BNST and CeA send modulatory neuropeptide (and inhibitory GABAergic) inputs to Orx-LH neurons, as well as inhibitory GABAergic projections to REM-Off regions (including vlPAG, LC, DRN, LPT). BLA neurons send excitatory glutamatergic projections to REM-Off neurons. REM-Off neurons inhibit SLD glutamate neurons that generate muscle atonia such as during REM under normal conditions. In NT1, with loss of Orx, there is an imbalance of excitatory/inhibitory input to REM-off regions. Inhibitory input from AMY to REM-Off neurons may be more pronounced as a consequence, resulting in less disinhibition (excitation) of SLD glutamate neurons. SLD glutamate neurons typically promote muscle atonia, and can lead to cataplexy upon being released from inhibitory control during wakefulness. Figure created using BioRender.com.

The circuitry underlying the switches between sleep and wake states is complex and involves multiple subregions, pathways, and neurotransmitters. However, given the interpretation of narcolepsy as an intrusion of REM sleep into wakefulness, and that episodes of cataplexy trigger a loss of muscle tone, as seen during REM sleep, it is important to review the circuitry believed to underlie the muscle atonia of REM sleep. During REM, it is believed that activation of the sublaterodorsal tegmental nucleus (SLD, also known as subcoeruleus nucleus) is what maintains muscle atonia. Downstream projections from the SLD ultimately lead to inhibition of motor neurons. During wake, projections from the Orx-expressing LH neurons to REM-regulating regions ultimately inhibit the SLD, allowing normal muscle tone to persist (for a detailed review of this circuitry, see Arrigoni et al. (2016)). In NT1, with loss of Orx neurons in the LH, this tonic inhibition of the SLD is lost, leading to inappropriate intrusion of muscle tone during wake (Figure 1).

Given the known role of the AMY in driving affective states, and the well-documented impacts of powerful emotions on sleep and wakefulness (Goldstein et al., 2013; Goldstein and Walker, 2014; ten Brink et al., 2022, 2023), decades of research have sought to elucidate the neural interactions between AMY circuits and Orx-LH neurons in NT1 (Adamantidis et al., 2020). Here, we (1) describe the distinct interactions of AMY subregions with hypothalamic and cortical systems relevant to narcolepsy, and their relationships to sleep regulation. Next, we (2) summarize descriptive studies in human patients that link changes in AMY activity and connectivity to NT1. In the following section, we (3) provide a synopsis of mechanistic studies in animal models of NT1 that causally demonstrate roles for the AMY in cataplexy bouts. Finally, we (4) synthesize these collective findings into an updated circuit model highlighting distinct AMY pathways in discrete contributions to affective regulation and emotional triggers in the context of narcolepsy with cataplexy.

## The amygdala, extended amygdala, and emotional arousal

### Amygdala and orexin interactions

Orexin is expressed specifically in neurons of the LH, and Orx-LH neurons have wide projections throughout the brain, including to structures of the AMY (Peyron et al., 1998). In particular, Orx-LH neurons richly innervate the BNST and CeA, where Orx exerts excitatory physiological effects that impact stress-related behaviors (Bisetti et al., 2006; Lungwitz et al., 2012; Weera et al., 2021). Anatomical studies in rodents confirmed that OX receptor mRNA is expressed in the AMY (Marcus et al., 2001) and orexinergic fibers are found in AMY subregions including the BNST, BLA, and CeA (Dustrude et al., 2018). There is a large literature on the effects of Orx/OX receptors in the AMY on stress and anxiety (Giardino and de Lecea, 2014; Yaeger et al., 2022a,b); however, many studies are in the context of social stress. It is unknown whether such negative socio-emotional states can potently trigger cataplexy in animal models of narcolepsy.

In a landmark study of Orx release in the human AMY, Blouin et al. used *in vivo* microdialysis to quantify the levels of AMY Orx during various behavioral and emotional conditions (Blouin et al., 2013). By implanting 8 epilepsy patients (who did not have narcolepsy) with probes targeted to the basal and lateral amygdalae (BA, LA), the authors showed that the higher concentrations of Orx in the AMY were reached during positive emotions (such as laughter), social interactions, negative emotions (frustration), and the onset of wake, whereas the lowest concentrations were during sleep (Blouin et al., 2013). These data illustrate that Orx release in the AMY is dynamic across sleep–wake arousal states and heightened emotional circumstances. Orx is highest during these emotional events, some of which are known to trigger cataplexy (laughing, frustration), so loss of AMY Orx release, such as in NT1, may have substantial impacts on how AMY neurons pass on their signals downstream in response to emotional stimuli (Figure 1).

In reciprocal fashion, Orx neurons do not only send axons that contact the AMY, but they also receive feedback from AMY neurons that input to the LH. Monosynaptic retrograde rabies virus mapping approaches revealed that neurons from the BNST and CeA both directly synapse onto the Orx-LH neuronal population (Gonzalez et al., 2016; Giardino et al., 2018). Given the complexity of the AMY circuitry, there are likely multifaceted roles for the AMY in mediating cataplexy and related effects of emotional stimuli on arousal. For example, discrete sets of BNST neuropeptide neurons can drive either approach or avoidance behaviors in mice *via* differential connectivity with LH-Orx neurons (Giardino et al., 2018), and CeA projections to the LH mediate aversion in rats (Weera et al., 2021).

Given the reciprocal projections between the AMY and Orx-LH neurons, and the critical role for AMY circuits in regulating mood and affect, our prevailing view is that loss of Orx tone, such as in NT1, would have profound consequences for AMY processing of emotional stimuli that could lead to increased liability for bouts of cataplexy. In this framework, the AMY comprises a central hub in which emotion-induced hyperactivity would stimulate the release of Orx, but in NT1, the absence of Orx release in the presence of elevated AMY activity would destabilize arousal and generate cataplexy (Figure 1). Thus, in the absence of Orx-LH neurons, BNST and CeA neuronal subpopulations likely have discrete contributions to cataplexy *via* their differences in neuropeptide release and distinct connectivity patterns with additional nodes in AMY and hypothalamic circuits (Giardino and Pomrenze, 2021).

### Amygdala and sleep circuit interactions

Beyond the dogmatic role for the AMY in mediating fear and anxiety responses, recent evidence supports the view that CeA, BLA, and BNST neurons can directly influence the balance between sleep and wake. For example, Ma et al. showed that optogenetic stimulation of neurotensin CeA neurons enhances non-REM (NREM) sleep (Ma et al., 2019), and Hasegawa et al. showed that optogenetic inhibition of dopamine receptor type-2 (Drd2) neurons in the BLA drives NREM-to-REM sleep transitions (Hasegawa et al., 2022). Going beyond these sleep-promoting functions, detailed contributions of CeA and BLA neurons to cataplexy have also been demonstrated in animal models of NT1, and are described in sections below. With regard to the *extended amygdala*, optogenetic stimulation of BNST GABA neurons can rapidly wake up mice from NREM sleep, and chemogenetic stimulation of BNST GABA neurons can extend wakefulness at the expense of both REM and NREM sleep (Kodani et al., 2017). Although yet unexplored, this evidence suggests that the BNST may also promote susceptibility to cataplexy in NT1 (Figure 1).

Further suggesting a role for the AMY in cataplexy, anatomical studies identified projections from the AMY onto regions important for regulating muscle atonia during REM sleep. Early studies in rats on projections to the REM-related regions of the pons (SLD) found cholera-toxin B (CTB) retrogradely labeled neurons in the CeA, as well as in the ventral and lateral regions of the BNST (Boissard et al., 2003). When double-stained for glutamate decarboxylase (GAD) many of these neurons were GAD-negative, indicating that they are likely not GABAergic (Boissard et al., 2003). In guinea pigs, CeA neurons were found to project to the dorsal part of the nucleus pontine oralis (which includes the SLD), and some of these stained positive for the vesicular glutamate transporter 2 (Vglut2), indicating their glutamatergic nature (Fung et al., 2011). More recent studies found that specifically GABAergic neurons of the CeA innervate the ventrolateral periaqueductal gray (vlPAG) and lateral pontine tegmentum (LPT) as well as the dorsal raphe nucleus (DRN) and locus coeruleus (LC), which are all thought to contribute to the generation of REM sleep (Burgess et al., 2013; Mahoney et al., 2017, 2019). One group also identified sets of bifurcating neurons that originate in the CeA and BLA and project to both the mPFC and vlPAG (Sun et al., 2019).

## The human amygdala in narcolepsy

The idea that the AMY is involved in narcolepsy with cataplexy originated nearly 60 years ago (Vizioli, 1964), yet the precise mechanisms underlying this relationship remain to be uncovered. Nevertheless, a growing body of work in human subjects—from psychophysical studies to neuroimaging analyses—has revealed increasingly clear evidence for dysfunction of several brain regions in NT1 (Wada et al., 2019), including the AMY.

In a study of metabolic changes in patients with narcolepsy, Poryazova et al. found that levels of the cell signaling transduction molecule myo-Inositol were reduced in the AMY of patients at rest, compared to controls (Poryazova et al., 2009b). Specifically among patients but not controls, these and other metabolite levels were also significantly negatively correlated with the levels measured in the hypothalamus and brainstem, indicating a narcolepsy-specific dysregulation of metabolic coordination between the AMY and other arousal-related brain circuits. At the anatomical level, multiple studies used structural MRI to calculate significantly reduced bilateral AMY volumes in NT1 patients compared to controls (Brabec et al., 2011; Kim et al., 2016).

Several neuroimaging studies in human patients using tomography, spectroscopy, or functional magnetic resonance imaging (fMRI) identified changes in amygdala activity or connectivity in the context of narcolepsy. For example, using SPECT imaging, Hong et al. identified increased blood flow to the right amygdala during episodes of cataplexy compared to normal wakefulness or REM sleep (Hong et al., 2006). Using fMRI, one study in children with NT1 demonstrated enhanced AMY activity during cataplexy triggered by watching a humorous video with no changes in AMY activity to laughter without cataplexy (Meletti et al., 2015). Interestingly, Vaudano and colleagues reported increased BOLD fMRI signal in the AMY of children and adolescent controls, but not NT1 patients, while laughing in response to a humorous video (Vaudano et al., 2019). In adult NT1 patients, AMY activity in response to humor was also enhanced relative to controls (Reiss et al., 2008; Schwartz et al., 2008). In another study, patients with NT1 showed higher AMY activity in response to winning a trial of a game when they were cued that they would receive a positive reward for winning before the trial (Ponz et al., 2010a), corroborating the idea that AMY activity increases in response to positive-valence stimuli. Further supporting this evidence indicating AMY hyperactivity, Huang et al. used fluorodeoxyglucose-PET imaging to identify hypermetabolic activity at rest in the AMY of teenage and young adult NT1 patients vs. controls (Huang et al., 2016). However, there are also conflicting reports of AMY activity in response to positive rewarding stimuli among NT1 patients. For example, Juvodden et al. reported increased AMY fMRI activation among NT1 patients compared to controls in response to neutral videos, but no group differences in AMY response to short humorous videos (Juvodden et al., 2019).

Across mammalian species, aversive auditory stimuli potentiate the Acoustic Startle Response (ASR; a reflexive muscle activity in response to a loud sound; Knudson and Melcher, 2016). This phenomenon is thought to be mediated in part by limbic structures, and lesions of the AMY disrupt the ASR in human patients (Angrilli et al., 1996). Strikingly, Khatami et al. observed that patients with narcolepsy also failed to show a potentiation of ASR by aversive stimuli, which led the authors to conclude that these individuals may have AMY dysfunction (Khatami et al., 2007). Consistent with these findings, Ponz et al. used fMRI to measure brain activity while subjects performed an aversive conditioning paradigm in which a visual cue served as a conditioned stimulus (CS+) that signaled a forthcoming mild electrical shock (Ponz et al., 2010b). While healthy controls showed significantly increased AMY activation in response to the CS+, this was dampened or absent in NT1 patients. Importantly, the authors demonstrated that there were no behavioral differences between groups in reaction times or task accuracy, nor in brain activity responses to the painful stimulus. These findings are important in indicating that NT1 patients do not only show AMY alterations in the context of positive emotional stimuli but may also indicate AMY dysregulation in the face of negative emotional stimuli.

More recent advances in analyses of “functional connectivity” allow researchers to use fMRI to identify pairs of brain regions that display synchronized (or desynchronized) changes in the time course of activity patterns. For example, Ballota et al. used resting-state fMRI in a small sample of adolescents to compare differences in functional connectivity between healthy controls and NT1 patients (Ballotta et al., 2021). Performing separate brain wide analyses with seed voxels placed in either the LH or AMY, they identified signatures of functional connectivity between the LH and hippocampus and between the AMY and posterior central gyrus/occipital regions that were significantly decreased in NT1. These findings indicate that loss of Orx-LH neurons in NT1 and corresponding dysfunction in domains of arousal, memory, and emotional processing may be associated with desynchrony of the LH and AMY.

Altogether, these data collected directly from the brains of human patients point toward a framework in which, relative to healthy controls, the AMY of NT1 patients is: smaller in volume, reduced in metabolic capacity (except during humor or cataplexy, when it may be hyperactive), deficient in responding to cues that predict aversive stimuli, and desynchronized from activity changes in posterior cortical regions.

## The amygdala in animal models of narcolepsy

Given the well-established role of the AMY in emotion processing and the fact that cataplexy is triggered by strong emotions, it is likely that the AMY plays a causal role in the pathophysiology of NT1. In foundational work, Jerry Siegel and colleagues observed profound loss of Orx axonal innervation in the AMY of narcoleptic dogs, providing some of the first evidence linking emotion-triggered cataplexy to AMY dysfunction (Siegel et al., 1999). Follow-up studies performed *in vivo* single-unit electrophysiological recordings of AMY neurons in narcoleptic canines across sleep–wake states, and during cataplexy (Gulyani et al., 2002). These authors identified two distinct subsets of neurons that were active during cataplexy: one subgroup that was also sleep-active (CeA, BLA), and one subgroup that was also wake-active (cortical amygdala; CoA). The authors proposed a mechanism in which cataplexy-active AMY neurons project to the BNST and LH, which pass information to the brainstem, ultimately relaying signals to pontine and medullary nuclei that suppress muscle tone (Gulyani et al., 2002).

In recent decades, focus shifted toward rodent models of cataplexy, particularly in mice (Scammell et al., 2009). Popular mouse models of cataplexy include: genetic deletion of Orx (knockout; KO), genetic deletion of both Orx receptors (OX receptor double knockout; DKO), or transgenic ablation of Orx-LH neurons (Orx-Ataxin3, Orx-DTA). In these models, exposure to appetitive stimuli (chocolate, wheel-running) can elicit bouts of EEG/EMG-defined cataplexy-like episodes (Burgess et al., 2013; Tabuchi et al., 2014). In addition to positive emotional stimuli, aversive stimuli like predator odor (an ethologically stressful stimulus to rodents) can also elicit cataplexy in these transgenic models of narcolepsy (Morawska et al., 2011; Hasegawa et al., 2014).

In one interesting study, viral overexpression of the Orx precursor gene (prepro-orexin) in CeA GABA neurons alleviated emotion-induced cataplexy in Orx KO mice (Liu et al., 2016). However, given that Orx is not normally expressed in cell bodies of the AMY, it is unclear how supraphysiological expression of Orx in these neurons would change their function to mitigate cataplexy. Later studies focused more on elucidating mechanisms of endogenous AMY neurocircuit function in the absence of Orx-LH neurons rather than attempting to introduce abnormal Orx expression in the AMY as a potential therapeutic approach.

In an activity mapping study from Oishi et al., investigators found that the number of cataplexy bouts following chocolate exposure in Orx KO mice was significantly positively correlated with the number of neurons demonstrating immunohistochemical labeling of the immediate early gene cFos in the BLA and posterior basomedial amygdala (pBMA; Oishi et al., 2013). These significant positive correlations were also observed with cFos expression in melanin-concentrating hormone (MCH) neurons of the LH, as well as the medial prefrontal cortex (mPFC) and olfactory cortices. The authors went on to show that anterior cingulate/prelimbic mPFC neurons send axonal projections to the medial division of the BLA (as well as to MCH-LH and Orx-LH neurons). Together, these findings suggest that loss of Orx neurons and exposure to positive emotional stimuli alters communication in the mPFC → AMY/LH pathways that increases liability to cataplexy.

Around the same time, Burgess et al. completed one of the first studies to directly manipulate the AMY to discern its role in cataplexy (Burgess et al., 2013). They first showed through viral tracing that GABAergic neurons in the CeA innervate multiple muscle atonia-associated brainstem regions including the vlPAG/LPT, LC, and DR (which provide inhibitory control over the SLD). Using the Orx KO model, mice were exposed to either positive stimuli (running wheel) or strong positive stimuli (running wheel + chocolate), with increased cataplexy bouts as the stimulus intensity increased. Excitotoxic lesions centered around the CeA decreased cataplexy in mice exposed to no stimuli, positive, or strong positive stimuli, suggesting a regulatory role for the CeA (Burgess et al., 2013). This led to a model in which under normal conditions, the AMY exerts an indirect inhibitory influence over the SLD, leading to normal muscle tone. In NT1, indirect inhibition of the SLD by the AMY is weakened, leading to loss of muscle tone (Figure 1).

In 2017, two independent groups using excitatory designer receptors exclusively activated by designer drugs (Gq-DREADDs) reported that chemogenetic stimulation of GABAergic CeA neurons in Orx KO mice increased the number of cataplexy episodes and the time spent in cataplexy (Mahoney et al., 2017; Snow et al., 2017). Interestingly, these manipulations did not alter overall sleep/wake times compared to non-manipulated Orx KO mice under baseline conditions (no salient stimuli) in either study. Using inhibitory Gi-DREADDs, experimenters also found that CeA-GABA inhibition was capable of reducing cataplexy in response to emotionally salient stimuli, but not under baseline conditions.

The authors went on to propose a mechanism in which GABAergic CeA neurons inhibit GABAergic neurons of the vlPAG/LPT, generating cataplexy *via* subsequent disinhibition of excitatory neurons in the brainstem subcoeruleus nucleus (also known as the SLD; Snow et al., 2017). This framework suggests that under healthy conditions, Orx-LH neurons provide excitatory inputs to vlPAG/LPT, but in NT1, loss of Orx inputs to these structures alters the excitatory/inhibitory balance, resulting in hyperactivity of the subcoeruleus nucleus.

Serotonergic neurons in the dorsal raphe nucleus (DRN-5HT) project to the AMY and receive Orx-LH input, suggesting their potential role in NT1. Hasegawa et al. (2017) showed that stimulation of DRN terminals in the AMY increased 5HT release and decreased cFos in the CeA/LA/BLA (reducing neural activity; Hasegawa et al., 2017). Optogenetic stimulation of this pathway in Orx-Ataxin3 mice decreased cataplexy-like episodes (CLEs). Similar to earlier work in Orx KO mice described above, these authors went on to show that Gq-DREADD excitation of the CeA in Orx-Ataxin3 mice also led to increased cataplexy, while Gi-DREADD inhibition of the CeA led to decreased cataplexy (Hasegawa et al., 2017). In this model, loss of Orx-LH neurons prevents DRN neuron activation, thereby reducing 5HT inhibition on the AMY. Resulting disinhibition leads to hyperactivity of the AMY ➔ brainstem pathways to generate muscle atonia.

To identify changes in patterns of neural activity in AMY neurons during cataplexy, Sun et al. performed *in vivo* recordings of CeA-GABA neurons of mice using fluorescent calcium imaging during both spontaneous cataplexy and emotion-induced cataplexy (predator odor; Sun et al., 2019). They identified subsets of neurons with low baseline activity that came online after emotion-induced cataplexy and became hyperactive in response to predator odor, providing additional evidence for AMY hyperactivity in NT1. Since these neurons were not hyperactive in response to odor in control mice, this may mean the AMY plays a modulatory role in the emotional processing of odor stimuli. Such hyperactivity in NT1 could send more inhibition onto key muscle-tone controlling brain regions like the vlPAG, resulting in cataplexy.

In a recent paper tying together AMY-Orx interactions with the mesolimbic dopamine (DA) reward system, Hasegawa et al. observed that DA release in the BLA was enhanced during chocolate feeding and leading up to cataplexy bouts (Hasegawa et al., 2022). They went on to use opto- and chemogenetic manipulations to demonstrate that inhibiting Drd2 neurons in the BLA induces cataplexy in Orx-Ataxin3 mice.

## Conclusion

Overall, the studies presented here provide clear evidence that the AMY is a key interface for the relationship between powerful emotional stimuli and cataplexy episodes in subjects with NT1. From studies conducted in human patients, scientists have collected important evidence showing dramatic changes of the AMY in individuals with NT1. For example, the AMY of NT1 patients is smaller in volume, deficient in responding to cues predicting aversive stimuli, desynchronized from other brain control networks, and displays dysregulated metabolic activity patterns in a state-dependent manner. However, some of these human studies lack sufficient subjects and/or do not test control subjects, and these would be important considerations for future progress in this field. While human work provides important correlative evidence showing dysregulation of emotion-induced activity of the AMY in NT1, animal models using advanced neurotechnologies and cell-type-specific approaches have revealed a more nuanced mechanistic understanding of the AMY’s role in cataplexy.

While there is only a small collection of publications directly examining the AMY in animal models of NT1, this rapidly growing literature provides major insights into the anatomical subregions, neuronal subpopulations, and circuit mechanisms that contribute to cataplexy. Importantly, most of these experiments investigated the effect of manipulating the AMY as a whole or large groups of heterogeneous neurons based on GABA expression. This may lead to the view that the AMY is exclusively inhibiting downstream regions, but the AMY also has glutamatergic neurons whose potential contributions to cataplexy have not yet been studied (Poulin et al., 2008; Vigneault et al., 2015; Ortiz-Juza et al., 2021). Additionally, emerging research on the CeA and BNST has revealed that cells can be characterized in a more nuanced way based on their molecular expression (Giardino and Pomrenze, 2021; O’Leary et al., 2022). Many subpopulations of AMY neurons that express classical neurotransmitters (like glutamate or GABA) also co-express other neuromodulators/neuropeptides, and these smaller clusters of cells may contribute uniquely to behavioral output (Ch'ng et al., 2018; Giardino et al., 2018). Future research into the role of the AMY in cataplexy could look at more specific cell types’ contribution. Transgenic mouse lines and newer genetically encoded sensors for neuropeptide/neuromodulator release may allow researchers to study the AMY in NT1 in this more nuanced way (Wu et al., 2022).

Unexplored as of yet is the role of reciprocal Orx-LH and AMY connections as it relates to cataplexy. OX receptors in the AMY mediate social stress-related behaviors (Yaeger et al., 2022a,b), though it is unknown whether these types of negative stimuli could trigger cataplexy. No studies have directly explored how the loss of AMY OX receptor signaling alters liability for cataplexy while animals are exposed to wheel-running, chocolate, predator odor, social stress, etc. While the model we show involves Orx-LH and AMY both converging on neurons that promote muscle tone, it will be beneficial to also understand how Orx-LH neurons may affect emotional processing in the AMY directly, since emotions can trigger cataplexy. In turn, it will be critical to understand how AMY → LH projections that might normally influence downstream muscle-atonia regulating regions would be altered in the absence of Orx (Figure 1). Viral tools and optogenetic/chemogenetic methods could be utilized to look at the contributions of specific pathways to this disorder.

One interpretation of NT1 as described earlier is that it is an intrusion of REM sleep into wakefulness (Adamantidis et al., 2020). We briefly review studies that have looked at the AMY’s unique role in sleep; however, little is known about how AMY neurons that receive Orx modulate REM sleep under normal and narcoleptic conditions. Greater understanding of how neurons of the AMY, particularly those that receive Orx inputs, may generate REM (Ma et al., 2019; Matei et al., 2022) and muscle atonia may reveal which circuits are most important to study further in the context of NT1. Studies completed thus far in both humans and animals have provided a good starting point for uncovering the AMY’s role in NT1, and future research may benefit from answering the additional gaps in our understanding described here.

## Author contributions

All authors listed have made a substantial, direct and intellectual contribution to the work, and approved it for publication.

## Funding

This work was supported by NIH grant AA025677-S1 (Diversity supplement for HS to grant number to 3R00AA025677-04S1) as well as NIH R00 AA025677 (WG), the Whitehall Foundation (WG), and a seed grant from the Stanford Center for Sleep and Circadian Sciences (WG).

## Conflict of interest

The authors declare that the research was conducted in the absence of any commercial or financial relationships that could be construed as a potential conflict of interest.

## Publisher’s note

All claims expressed in this article are solely those of the authors and do not necessarily represent those of their affiliated organizations, or those of the publisher, the editors and the reviewers. Any product that may be evaluated in this article, or claim that may be made by its manufacturer, is not guaranteed or endorsed by the publisher.
